# Rare emergence of delamanid resistance in multidrug-/rifampicin-resistant tuberculosis patients receiving delamanid-containing regimens: a prospective multicentric study in China

**DOI:** 10.1186/s12941-025-00820-9

**Published:** 2026-07-02

**Authors:** Wei Shu, Qingshan Cai, Yuping Huang, Yuanhong Xu, Yuanyuan Shang, Ruixia Liang, Liang Yan, Lin Wang, Long Jin, Yi Pei, Zhongfeng Huang, Shanshan Li, Yufeng Wang, Mengqiu Gao, Liang Li, Yu Pang

**Affiliations:** 1 Clinical Center on TB, Beijing Chest Hospital, Capital Medical University/ Beijing Tuberculosis & Thoracic Tumor Research Institute, Beijing, P.R. China; 2 Department of Tuberculosis, Hangzhou Red Cross Hospital, Zhejiang Tuberculosis Diagnosis and Treatment Center, Hangzhou, P.R. China; 3 Department of Laboratory Medicine, Chest Hospital of Guangxi Zhuang Autonomous Region, Liuzhou, P.R. China; 4 Department of Laboratory Medicine, Chengdu Public Health Clinical Center, Chengdu, P.R. China; 5 Department of Bacteriology and Immunology, Beijing Key Laboratory on Drug-Resistant Tuberculosis Research, Beijing Chest Hospital, Capital Medical University/ Beijing Tuberculosis & Thoracic Tumor Research Institute, Beijing, P.R. China; 6 Tuberculosis Department, Henan Chest Hospital, Zhengzhou, P.R. China; 7 Department of Laboratory Medicine, Jiangxi Chest Hospital, Nanchang, P.R. China; 8 Department of Laboratory Medicine, The Third People’s Hospital of Kunming, Kunming, P.R. China; 9 The Eight Wards of Tuberculosis Internal Medicine, Infectious Hospital of Heilongjiang Province, Harbin, P.R. China; 10 Department of Tuberculosis, Changsha Central Hospital, Changsha, P.R. China; 11 The Drug-Resistant Tuberculosis Division, Guiyang Public Health Treatment Center, Guiyang, P.R. China; 12 Department of Laboratory Quality Control, Innovation Alliance on Tuberculosis Diagnosis and Treatment (Beijing), Beijing, P.R. China; 13 Department of Tuberculosis, Beijing Chest Hospital, Capital Medical University/Beijing Tuberculosis & Thoracic Tumor Research Institute, Beijing, 101149 P.R. China

**Keywords:** Tuberculosis, Delamanid resistance, Multidrug-/Rifampicin-resistant

## Abstract

**Background:**

Delamanid (DLM) is a promising drug recommended for treatment of multidrug-/rifampicin-resistant tuberculosis (MDR/RR-TB). However, there are very little data about the emergence of drug resistance in patients with the use of DLM-containing regimens. The study aimed to monitor the dynamics of susceptibility of MTB to DLM and investigate the potential mechanism conferring decreased susceptibility.

**Methods:**

A prospective cohort study was conducted, enrolling MDR/RR-TB patients with culture-confirmed diagnoses across 10 study sites. Serial sputum samples were collected from participants receiving DLM-containing regimens at baseline (treatment initiation), week 2, week 4, and every 4 weeks thereafter until treatment completion. The in vitro susceptibility of *Mycobacterium tuberculosis* isolates to DLM was assessed using the BACTEC MGIT 960 system.

**Results:**

A total of 263 MDR/RR-TB patients were included from 2020 to 2024 in the present study, favorable outcomes were recorded in 187 patients (71.1%). The distribution of minimal inhibit concentration (MIC) for a bacterial population to DLM was unimodal, and most isolates tested had a MIC value of < 0.015 µg/mL. The MIC_50_ and MIC_90_ of MTB isolates were 0.03 and 0.06 µg/mL, respectively. Using the 0.12 µg/mL as a proposed epidemiological cutoff value, the resistance to DLM was found in 0.76% (2/263) of MTB isolates and no mutations were identified within loci conferring DLM resistance. Additionally, the remaining 81 (30.8%) with serial isolates were included in our analysis. 70 out of 81 patients exhibited no change in DLM MICs. In contrast, the reduced susceptibility to DLM was noted in 11 patients, defined as no less than 2-fold increase in MIC value compared with that of baseline.

**Conclusions:**

Primary DLM resistance was rare (0.76%), and resistance emergence during treatment was infrequent, though some cases of reduced susceptibility were observed.

## Introduction

Tuberculosis (TB), caused by *Mycobacterium tuberculosis* complex (MTBC), remains a major global public health challenge. According to the latest World Health Organization (WHO) estimates, TB approximately 10.8 million individuals and resulted in 1.25 million deaths in 2024 [1, 2]. Of particular concern is multidrug-resistant TB (MDR-TB),, defined as resistance to both rifampicin and isoniazid, which complicates treatment for an estimated 410,000 cases annually. The global treatment success rate for MDR-/rifampicin-resistant TB (MDR/RR-TB) remains suboptimal at 68%, largely due to the limited efficacy of second-line anti-TB regimens [1]. Within the Western Pacific region, the treatment success rate for MDR/RR-TB was reported at 70%, while China only achieved a success rate of 66% [1]. Thus, there is an urgent need for development of innovative strategies for treatment to tackle the high rates of treatment failure and mortality in MDR/RR-TB patients [3, 4].

Delamanid (DLM) is a promising bicyclic 4-nitroimidazole drug that represents one of only three novel drugs recommended for treatment of MDR/RR-TB in recent years [5]. Previous preclinical studies have demonstrated that drug has potential to shorten and simplify the treatment of drug-resistant TB [6, 7]. In adults with MDR-TB, DLM plus an optimized background regimen resulted in a significant increase in sputum culture conversion compared with control group after 2 months of therapy (45.4% versus 29.6%) [8]. In a recent meta-analysis, 591 patients undergoing DLM-containing regimens achieved a success rate of 80.9% [9], emphasizing the promising efficacy of DLM in the treatment of drug-resistant TB. Unfortunately, the emergence of antimicrobial drug resistance comprises the major risk that threatens effective management of public health infectious diseases globally [10, 11]. DLM resistance has been reported in the context of inadequate therapy regimens [12]. In vitro experiments also demonstrate a spontaneous resistance frequence of 9.0 × 10^− 6^ for H37Rv strain after exposure to DLM [13]. The surprisingly rapid acquisition of DLM resistance highlights the need for appropriate use of this new drug and underlines the importance of drug resistance surveillance [14]. However, due to the complexity related to proper drug susceptibility testing, there are very little data regarding the emergence of drug resistance in patients with the use of DLM-containing regimens.

China is one of hotspots of MDR/RR-TB worldwide, accounting for 7.3% of global burden [1]. In 2018, National Medical Products Administration of China (NMPA) conditionally approved DLM as part of combination therapy to adults with a definite diagnosis of MDR/RR-TB (Delamanid Tablets, NMPA Approval Number HJ20181244). Post-marketing study was initiated in China in 2020 to assess the efficacy and safety of DLM in TB patients. Based on this nationwide clinical trial, we conducted DLM-resistance surveillance of MDR/RR-TB cases to determine the proportion of DLM resistance in the Chinese cohort without prior exposure. We also aimed to monitor the dynamics of susceptibility of MTB to DLM and investigate the potential mechanism conferring decreased susceptibility.

## Materials and methods

### Study design and collection site

A prospective cohort study was conducted by inclusion of MDR/RR-TB patients with positive cultures in 10 sites, including Beijing Chest Hospital, Capital Medical University, Hangzhou Red Cross Hospital, Guangxi Zhuang Autonomous Region Chest Hospital, Chengdu Public Health Clinical Center, Hennan Provincial Chest Hospital, Jiangxi Chest Hospital, Kunming Third People’s Hospital, Infectious Disease Hospital of, Heilongjiang Province, Changsha Central Hospital, Guiyang Public Health Treatment Center.

### Sample collection

We obtained sputum samples from these patients undergoing therapy with DLM at entry, 2, 4 and every 4 weeks from treatment completed to commencement of treatment. Specimens were submitted to local laboratory for mycobacterial culture with BACTEC MGIT 960 system (Becton Dickinson) according to clinical routine procedures of each site. All positive culture were stored in 7H9 medium supplemented with 10% of oleic acid-albumin-dextrose-catalase (OADC) and 5% glycerol, and stored in -70℃ for further in vitro susceptibility testing purpose.

### Drug susceptibility testing for Delamanid

The in vitro susceptibility of MTBC isolates to DLM was determined with the BACTEC MGIT 960 as recommend by the WHO’s guidelines [15]. Briefly, we scaped the 4-week-old fresh colonies from the Lowenstein-Jensen (L-J) slant. After vigorous mixing for 1 min on a vortex mixer, a suspension of MTB isolate was adjusted to McFarland 0.5 by adding sterile saline. The turbidity of the suspension was measured with a routine suspension turbidity meter. 0.5 ml of the test strain suspension was inoculated into the MGIT tubes supplemented with 0.8 ml of supplement and 0.1 ml of the corresponding drug solution. For preparation of the drug-free growth control tube, the strain suspension was diluted 1:100 with sterile saline, and then 0.5 ml was pipetted into the tube. Minimum inhibitory concentration (MIC) of DLM was done by testing at 5 concentrations in separated tubes, ranging from 0.015 to 0.12 µg/ml and the control tubes. The susceptibility testing sets were incubated into the MGIT 960 instrument and continuously monitored automatically. Results were interpreted as follows: at the time when the growth unit (GU) of the drug-free control tube reached 400, the MIC in MGIT was defined as the lowest drug concentration that kept growth < 100 GU. DLM resistance was declared if the strain had a MIC > 0.12 µg/ml.

### DNA extraction and sequencing

The genomic DNA of MTB strain was extracted using cetyl-trimethylammonium bromide (CTAB) method as previously described [16]. The DNA was used as template to amplify and sequence the gene fragment conferring DLM resistance, including DLM prodrug activation (ddn and fgd1) and F420 biosynthetic pathway (fbiA, fbiB, fbiC). The primers used herein followed our previous study [13]. The 50 µL PCR reaction mixture contained 5 µL 10×PCR buffer, 200 µM each dNTP, 0.2 µM each primer set, 1 µL of template DNA, and 1 U HotStar Taq polymerase (Qiagen). The PCR cycling was performed according to the procedures reported by our group. The amplification products were sent to Tianyihuiyuan Company (Beijing, China) for DNA sequencing service. The DNA sequences were aligned with the reference sequences of H37Rv strain (ATCC 27294) using BioEdit Version 7.1.3 software.

### Statistical analysis

Data were analyzed as reported previously according to Clinical and Laboratory Standards Institute guidelines [17].The epidemiological cutoff value (ECV) for DLM resistance was defined as the representative MIC value for at least 95% of wild-type isolates within the mixed population. All statistical description were performed using Excel version 365.

## Results

### Study population

Overall, a total of 263 MDR/RR-TB patients were included in the present study. Baseline demographic and clinical characteristics are summarized in Table 1. The median age of patients at enrollment was 40 ± 13.1 years. 62.4% of participants were male; and nearly one third had a low body mass index (BMI) < 18.5 kg/m². Most patients 72.2% (190/263) included had previous anti-TB treatment history. Additionally, FQ and amikacin resistance were noted in 38.5% and 14.6% of these participants, respectively.

**Table 1:** Characteristics of the participants

Variable	Total(*N* = 263)
Age-year-Mean ± SD	40 ± 13.1
Age- no. (%)	
< 40	144(54.7)
40–59	98(37.3)
> 60	21(8.0)
Male sex- no. (%)	164(62.4)
Baseline BMI, kg/m²-Mean ± SD	20.5 ± 4.0
Low Baseline BMI- no. (%)	77(29.3)
Previous anti-TB treatment history- no. (%)	190(72.2)
Alanine aminotransferase, U/L-Meian(Q1-Q3)	12(7.2–19.0)
Total Bilirubin, umol/L-Meian(Q1-Q3)	7.5(5.3–10.1)
Fasting Plasma Glucose, mmol/L-Meian(Q1-Q3)	4.9(4.5–5.7)
Blood uric acid, umol/L-Meian(Q1-Q3)	338.0(254.5-452.4)

*Low BMI:* BMI < 18.5 kg/m²

### MIC distribution at baseline

**Fig. 1:**
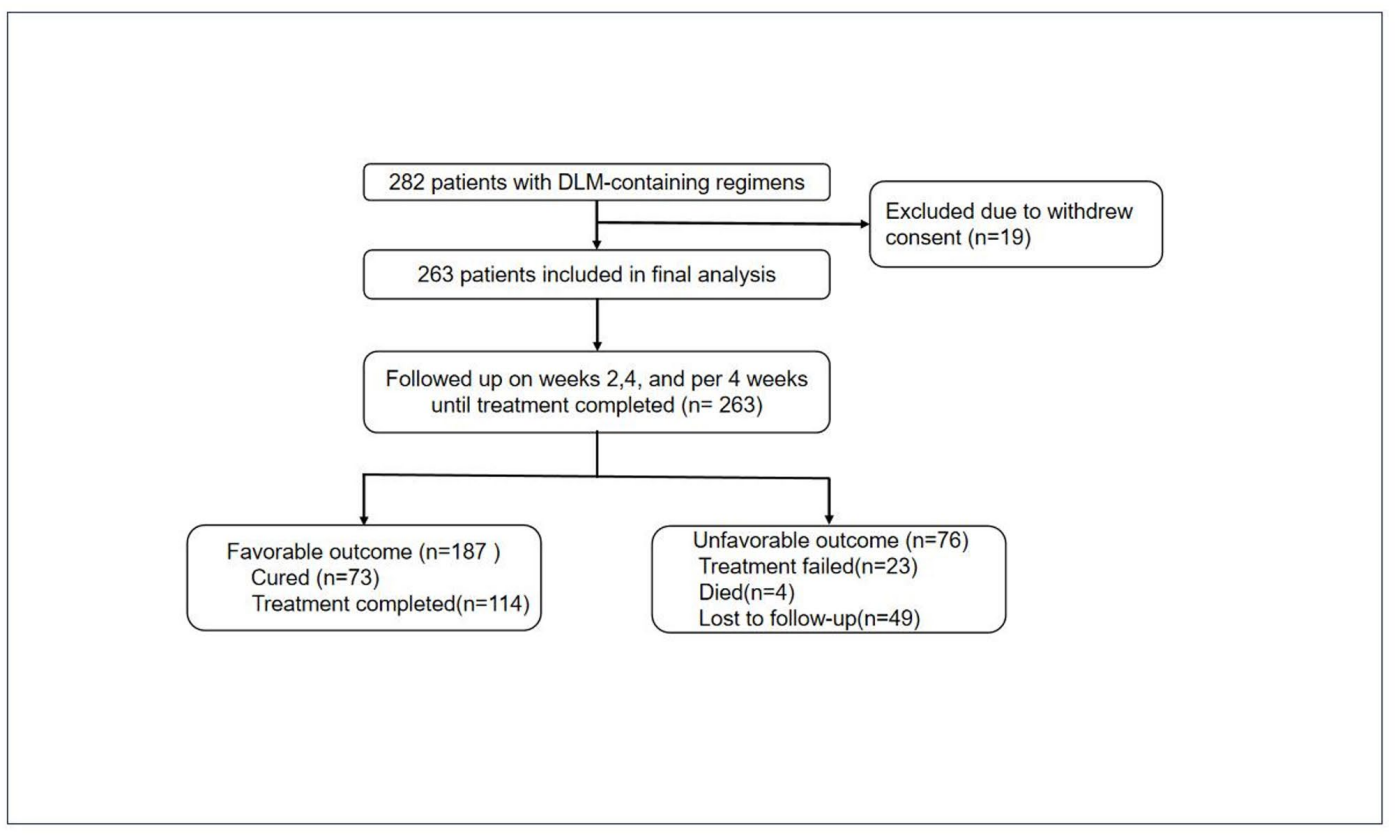
Participant enrollment

**Fig. 2:**
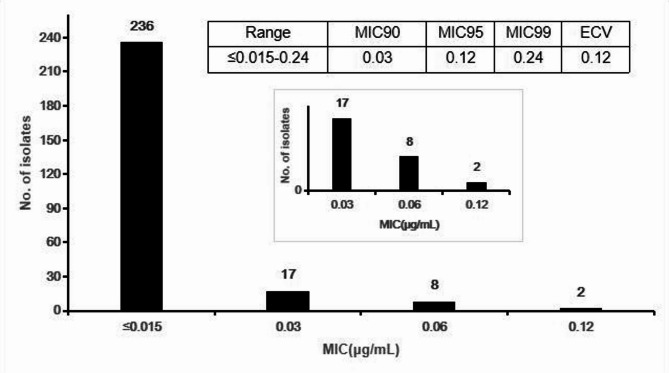
Distribution of multidrug-resistant tuberculosis isolates with varying. MIC values. *ECV* epidemiological cutoff value, *MIC* minimal inhibitory concentration

Treatment outcomes of patients receiving DLM-containing regimens are shown in Fig. 1. Of 263 MDR/RR-TB patients completing treatment course, favorable outcomes were recorded in 187 patients (71.1%); whereas the remaining 76 (28.9%) had unfavorable outcomes. Finally, these patients with positive cultures were further included for in vitro BLM susceptibility testing. As demonstrated in Fig. 2, the distribution of MICs for a bacterial population to DLM was unimodal, and most isolates tested had a MIC value of < 0.015, indicating that tubercle bacilli were innately susceptible to DLM. The MIC_50_ and MIC_90_ of MTB isolates were 0.03 and 0.06 µg/mL, respectively. Using the 0.12 µg/mL as a proposed ECV, the resistance to DLM was found in 0.76% (2/263) of MTB isolates. Notably, these two patients with DLM MIC bordering breakpoint completed the treatment course and achieved culture conversion by the end of treatment. However, no mutations were identified within loci conferring DLM resistance (Table 2).

**Table 2:** Demographic and clinical characteristics of patients with initial Delamanid resistance

Patient ID	Sex	Age, y	Patient Category	Drug Susceptibility	Minimum Inhibitory Concentration (µg/mL)		Sputum Culture Conversion	Clinical Outcome
					Baseline	Last		
dE0043	F	41	New	MDR	0.12	0.12	8 weeks	Treatment completed
dF0001	F	21	Previously treated	MDR	0.12	0.12	4 weeks	Treatment completed

*F* female, *M* male, *MDR* multidrug-resistant

### Change in DLM mics after DLM exposure

**Fig. 3:**
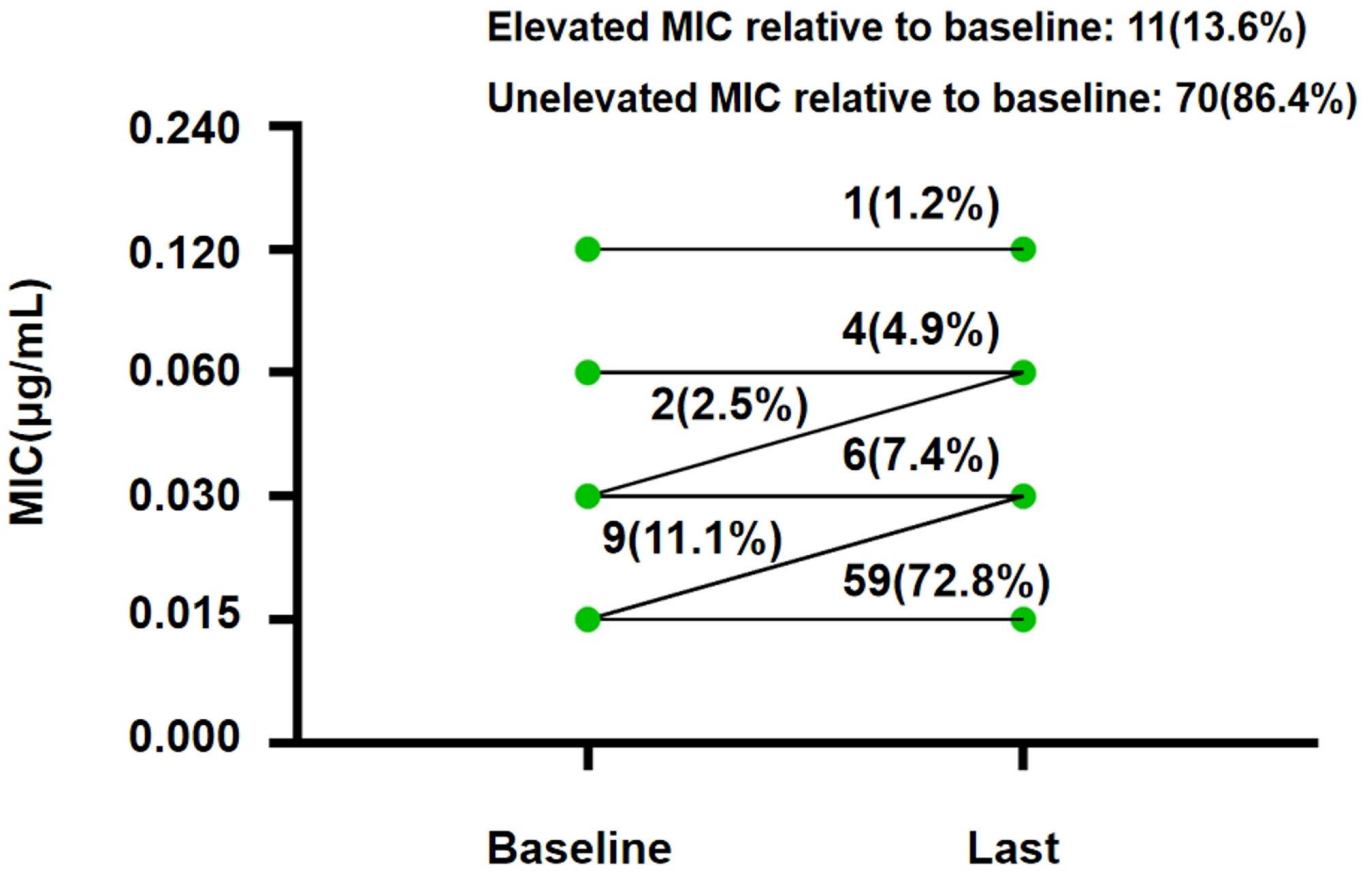
Dynamic change in DLM MIC

We also assessed the dynamic change in DLM MICs after DLM exposure. Of 263 participants, 182 (69.2%) achieved culture conversion within 2 weeks; thus, the remaining 81 (30.8%) with serial isolates were included in our analysis. As illustrated in Figs. 3 and 70 out of 81 patients exhibited no change in DLM MICs over the study period. In contrast, the reduced susceptibility to DLM was noted in 11 patients, defined as no less than 2-fold increase in MIC value compared with that of baseline. Interestingly, the MICs of isolates with reduced DLM susceptibility were below the breakpoint, yielding 0.03 µg/mL and 0.06 µg/mL for 9 (11.1%) and 2 (2.5%) isolates, respectively.

## Discussion

The emergence of drug-resistance has emerged as one of the major challenges for TB control, which results in the rapid loss of the new drugs, such as delamanid and bedaquiline [11, 18]. The systematic surveillance of initial resistance is of great importance for optimal use of DLM in drug-unexposed individuals. In this study, we performed a prospective study to evaluate the DLM resistance in the Chinese population. Our data demonstrated that initial resistance occurs in 0.76% (2/263) of MDR-TB patients. In a multicenter phase II trial on MDR-TB patients, DLM resistance was noted in 0.63% (2/316) participants at baseline [8]. Similarly, DLM baseline resistance was observed in 0.39% (2/511) of participants in a phase III trial [19]. Recently, the in vitro DLM susceptibility of 420 clinical isolates collected in Korea, DLM resistance was noted in 41 (9.8%) isolates with microdilution method [20]. Using the same methodology, another in vitro study on 220 clinical MDR-TB isolates from DLM-naïve patients in China identified 7 (3.18%) DLM-resistant isolates [21]. Obviously, the DLM baseline resistance rates of these latter studies were significantly higher than that in our study, and in phase II and III study. There are at least two possible explanations for the observed geographic differences in the proportions of resistance. A recent molecular epidemiological study has identified a strong lineage effect on the MIC of MTB isolates. Hence, one potential explanation for this observed difference may thus reflect the potential geographic diversity in the proportions of DLM resistance of circulating isolates [22]. In addition, we found that the broth microdilution method was used in these latter studies versus liquid culture MGIT method in our study. At present, the MGIT 960 system is proposed as the reference method for DLM DST. Although these two methods agreed in general for some drugs [23, 24], we hypothesize that there is a tendency for the microdilution method to overestimate the MIC values of DLM. As highlighted here, DLM is a great promising anti-TB drugs for the therapy of MDR/RR-TB patients considering that DST is currently not widely implemented in China.

Another interesting finding of this study was that we recorded rare emergence of DLM resistance in these patients receiving DLM-containing regimens. In a retrospective clinical study in China [25], 11.7% (11/94) MDR-TB patients treated with BDQ-based regimens exhibited reduced bedaquiline susceptibility, 5 (1.8%) of which achieved criteria for acquired resistance, indicating that MDR-TB patients had higher rate of acquired bedaquiline resistance compared to that of DLM. The emergence of resistance depends in part on high mutational rate of tubercle bacilli, leading to the selection of resistant isolates during suboptimal chemotherapy [26]. However, our previous in vitro experimental studies revealed that the average mutation rate for DLM resistance was 1.0*10^− 5^, which was significantly higher than that for BDQ (7.09 × 10^− 8^) despite the existence of intraspecific difference in MTB isolates [13, 27]. The obvious difference between the in vitro and in vivo observations on the pattern of emergence of drug resistance could be explained by several possible mechanisms. On the one hand, the in vitro model majorly focuses on fast-replicating bacteria, whereas a large fraction of the slow-replicating bacterial population is neglected, which may result in biased estimation of spontaneous mutation rate. In line with our hypothesis, a previous study by Bergval and coresearchers found that the in vivo mechanism of isoniazid resistance is not reflected by in vitro experiments [28]. On the other hand, the use of effective drug combinations is an important approach to decrease the emergence of drug resistant bacteria while increasing the therapy efficacy. In our cohort, approximate 1.5%(4/263) of patients underwent treatment with DLM and BDQ, which may be another mechanism of rare emergence of DLM resistance. Apparently, this leads to high bactericidal activity against tubercle bacilli, so the bacteria with single mutation conferring alternative drug could be killed by the partner drug. Future research is warranted to uncover the mechanism involving in this phenomenon.

We also acknowledged several obvious limitations to our study. First, all experiments for MIC determination were not performed in triplicate. This would further produce the uncertainty introduced by systematic error. Second, due to the remarkable bactericidal effect of DLM, as well as linezolid and bedaquiline, the majority of patients achieved culture conversion within 1 month, further preventing us to dynamically monitoring DLM susceptibility during the treatment course. The small sample size of patients with MIC shifts also hampered us to investigate the correlation between sub-breakpoint MIC shifts and treatment outcomes. Third, it is noteworthy that several participants had a persistent positive sputum culture after 6 months of treatment. However, we did not record the increased MIC values and the emergence of DLM resistance. Our results suggest that an unknown mechanism is also associated with failure in controlling bacterial multiplication. Fourth, only classic loci conferring DLM resistance were sequenced in the present study, which could not comprehensively identify all potential mutation types. Further application of the whole genome sequencing is of importance to delicate the novel molecular mechanism of DLM resistance. Despite these limitations, the present study provides important insights into clinical application of DLM in the treatment of MDR/RR-TB patients.

To conclude, our data show that initial DLM resistance is present in only 0.76% of MDR/RR-TB patients. Resistance emergence during treatment with DLM-containing regimens was also rare. These findings support delamanid as a highly promising option for the treatment of MDR/RR-TB.

## Data Availability

No datasets were generated or analysed during the current study.
